# CEA Decline Predicts Tumor Regression and Prognosis in Locally Advanced Rectal Cancer Patients with Elevated Baseline CEA

**DOI:** 10.7150/jca.49252

**Published:** 2020-09-23

**Authors:** Zerong Cai, Lingyu Huang, Yufeng Chen, Xiaoyu Xie, Yifeng Zou, Ping Lan, Xiaojian Wu

**Affiliations:** 1 Department of Colorectal Surgery, the Sixth Affiliated Hospital, Sun Yat-sen University, Guangzhou, Guangdong, China; 2 Department of Medical Oncology, the Sixth Affiliated Hospital, Sun Yat-sen University, Guangzhou, Guangdong, China; 3 Guangdong Institute of Gastroenterology, Guangzhou, Guangdong, China; 4 Guangdong Provincial Key Laboratory of Colorectal and Pelvic Floor Diseases, Guangzhou, Guangdong, China

## Abstract

**Purpose:** To investigate the value of carcinoembryonic antigen (CEA) decline in predicting pathological tumor regression and outcome for locally advanced rectal cancer (LARC) patients who received neoadjuvant therapy with elevated baseline CEA.

**Methods:** LARC patients with elevated pre-treatment CEA who received neoadjuvant therapy and radical tumor resection were retrospectively collected. Serum CEA level during treatment were recorded and the predictive value of pre-treatment CEA, post-treatment CEA and CEA ratio (CEA^post-treatment^ /CEA^pre-treatment^) for tumor regression grade (TRG), overall survival and diseases free survival were estimated by logistic regression or cox proportional hazard regression.

**Results:** Two hundred and eighty-four LARC patients with elevated pre-treatment CEA were enrolled and the baseline, post-treatment CEA level and CEA ratio were 11.87 (5.02-731.31) ng/ml, 4.23 (0.50-173.80) ng/ml and 0.31(0.01-2.55) respectively. CEA level in 59.2% of the patients declined to normal after neoadjuvant therapy. Multivariate analysis showed that CEA ratio was an independent predictor for TRG (OR=3.463, 95% CI: 1.269-9.446, *P=*0.015) and tumor downstage (OR=0.393, 95% CI: 0.187-0.829, P=0.014). Patients with normalized post-treatment CEA level had better overall survival (*P*=0.010) and disease free survival (*P*=0.003) than those with elevated CEA level. Higher post-treatment CEA was an independent unfavored predictor for overall survival in LARC patients with elevated pre-treatment CEA (OR=1.042, 95% CI: 1.017-1.067, *P*=0.001).

**Conclusion:** Post/pre-treatment CEA ratio predicted tumor regression in term of TRG and tumor downstage for LARC patients with elevated pre-treatment CEA and higher post-treatment CEA predicted poor overall survival.

## Introduction

Rectal cancer was one of the key malignancies which endangered human health. For patients with locally advanced rectal cancer (LARC), neoadjuvant therapy was the standard treatment before surgery, which could improve the local control, increase the sphincter preservation rate and decrease local recurrence^1^. Predicting tumor regression could contributed to choose individual post-operative chemotherapy reagents and helped precise surgical management including transanal local excision or “wait and see” strategy^2^, ^3^. Several predictive models including tumor volume reduction rate^4^, parameters of magnetic resonance imaging (MRI)^5^, ^6^, molecular marker clusters^7^ were reported to accurately predict tumor regression of LARC by neoadjuvant chemotherapy. However, the complexity restricted their clinical practicality.

CEA was a widely recommended serum tumor marker for rectal cancer and was associated with tumor stage, differentiation grade and distant metastasis^8^. We previously categorized CEA into quintiles and it was showed that preoperative CEA quintile was an independent predictor of unfavorable prognosis in colorectal cancer patients even within normal range^9^. Nevertheless, the value of CEA change during neoadjuvant treatment in predicting neoadjuvant therapy response and patients' prognosis still remained unclear. Researches by Hu H 10 and Kim J.Y^11^ showed that LARC patients with exponential CEA decrease were more likely to achieved pathological complete response after neoadjuvant therapy. However, those CEA decrease patterns were still too complicated to be determined and the sample sizes were relatively small.

Therefore, we retrospectively collected LARC patients with elevated pre-treatment CEA who received neoadjuvant therapy and radical tumor resection, to investigate the predictive value of CEA change during neoadjuvant therapy for tumor regression and prognosis.

## Methods

### Patients and Characteristics

We retrospectively collected LARC patients who received neoadjuvant chemotherapy with or without radiotherapy and underwent low anterior rectal resection or abdominoperineal excision plus total mesorectal excision from January 1, 2010 to December 31, 2017 in the Sixth Affiliated Hospital of Sun Yat-Sen University. Eligible criteria was:(1) Histologically diagnosed primary rectal adenocarcinoma with baseline serum CEA level higher than 5ng/ml; (2) Clinical diagnosed with resectable T3-4 or N+ tumor without distant metastatic disease by pelvic contrast-enhanced MRI and chest-abdominal-pelvic contrast enhanced computed tomography (CT) scan; (3) Received 3-7 cycles of neoadjuvant chemotherapy with or without radiotherapy before surgical treatment. Exclusion criteria was: (1) With history of other malignant disease within previous 5 years; (2) Received inadequate (<3 cycles) pre-operative chemotherapy because of any reason such as intolerable toxicity or uncontrolled digestive hemorrhage or perforation; (3) Recurrent rectal cancer; (4) Loss of serum CEA level data during neoadjuvant treatment.

Serum CEA level was measured by Architect CEA Reagent Kit (Abbott Laboratories. US) within two weeks before neoadjuvant chemotherapy (pre-treatment CEA) and within one week before surgery (post-treatment CEA). Elevated CEA level was defined as >5ng/ml^12^, ^13^.

All patients underwent fluorouracil or capecitabine-based neoadjuvant chemotherapy for 3-7 cycles with or without radiotherapy and following curative surgery. Neoadjuvant chemotherapy regimens included: fluorouracil/leucovorin or capecitabine alone, fluorouracil/leucovorin or capecitabine plus oxaliplatin, fluorouracil/leucovorin plus irinotecan. For patients who received pre-operative radiotherapy, radiotherapy was delivered at 1.8-2.0 Gy daily for a total of 23-28 fractions over 5 to 6 weeks and a total dose of 46.0-50.4 Gy.

All resections specimens were examined according to the TNM classification in the AJCC Cancer Staging Manual^14^. Tumor regression was determined by tumor regression grade (TRG) and tumor downstage. TRG 0/1/2/3 was defined according to NCCN protocol^15^. Tumor downstage was defined by pathological TNM stage < clinical TNM stage. Prognosis information including overall survival and disease free survival was collected from the Follow-up Office of the Sixth Affiliated Hospital of Sun Yat-sen University. High standard of ethics was applied in carrying out the investigation in compliance with the Helsinki Declaration and under the approval of the institutional review board ethics committee of the Sixth Affiliated Hospital of Sun Yat-sen University (No. L2015ZSLYEC-041).

### Statistical analysis

Statistical analysis was performed by IBM SPSS software (Version19.0) or GraphPad Prism software (Version 7.0.4). Continuous variables were presented as mean±standard deviations (SD) or median (maximum-minimum) and categorical data were present as number (percentage).* T*-test or Mann Whitney-U test were used to compare the continuous variables and categorical variables were compared by chi-square test. Logistic regression was used to estimate predictors for TRG and tumor downstage. Receiver-Operating Characteristic (ROC) analysis was carried out for obtaining the area under the curve (AUC) and the best cut-off value was calculated, corresponding to the highest Youden index. Kaplan-Meier method and Log-rank test was used to calculate and compare cumulative survival rate. Cox proportional hazard regression model was used to estimate risk factors for the prognosis. *P* value <0.05 was considered statistically significant.

## Results

### Patient's characteristics

Two hundred and eighty-four patients with elevated pre-treatment serum CEA level were enrolled and the characteristics are summarized in Table 1. Among them, 200 cases (70.4%) of patients were male gender and the median age was 59 (20-81). Twenty-three cases (8.1%) of patients had comorbidity of diabetes mellitus. According to the pre-treatment CT and MRI, 37 cases (13.8%) of cTNM stage II and 231 cases (86.2%) of cTNM stage III were diagnosed. 39.2% (n=107) of them received neoadjuvant radiotherapy. The median pre-treatment of serum CEA level of patients were 11.87 (5.02-731.31) ng/ml and declined to 4.23 (0.50-173.80) ng/mL after neoadjuvant treatment, with a median post/pre-treatment CEA ratio of 0.31 (0.01-2.55).

### Post-treatment CEA level and post/pre-treatment CEA ratio were associated with tumor regression grade and tumor downstage after neoadjuvant chemotherapy

One hundred and ten patients (38.7%) achieved TRG 0/1 and 185 patients (65.1%) achieved tumor downstage. As showed in Table 1, patients who achieved TRG 0/1 had lower post-treatment serum CEA level (2.77 vs 4.91, *P<*0.001) and CEA ratio (0.23 vs 0.37, *P<*0.001) when compared with those achieved TRG 2/3. When in terms of tumor downstage, patients who achieved tumor downstage were characterized with lower post-treatment CEA level (3.75 vs 4.51, *P=*0.043) and lower CEA ratio (0.28 vs 0.38, *P=*0.025), when compared with those did not.

### Low CEA ratio predicted TRG 0/1 and tumor downstage in LARC patients

As showed in Table 2 and Table 3, multivariate logistic regression model suggested that lower post-treatment CEA (OR=0.936, 95% CI: 0.883-0.993, *P=*0.027) and lower CEA ratio (OR=0.289, 95% CI: 0.089-0.495, *P<*0.001) were both TRG 0/1 predictive factors. Tumor downstage predictive factors included older age (OR=1.031, 95% CI: 1.008-1.054, *P=*0.007), cTNM stage III (OR=4.405, 95% CI: 2.150-9.025, *P<*0.001), G1/2 tumor differentiation (OR=2.757, 95% CI: 1.238-6.137, *P=*0.013) and lower CEA ratio (OR=0.393, 95% CI: 0.187-0.829, *P=*0.014). We performed receiver operating characteristic (ROC) curve analysis and it was suggested that the best cutoff value of CEA ratio for predicting TRG 0/1 was 0.23, and for predicting tumor downstage the best cutoff of CEA ratio was 0.53 according to the highest Youden index.

### Higher post-treatment CEA level predicted unfavored overall survival and higher pre-treatment CEA level predicted unfavored disease free survival in LARC with elevated pre-treatment CEA

**Figure 1:**
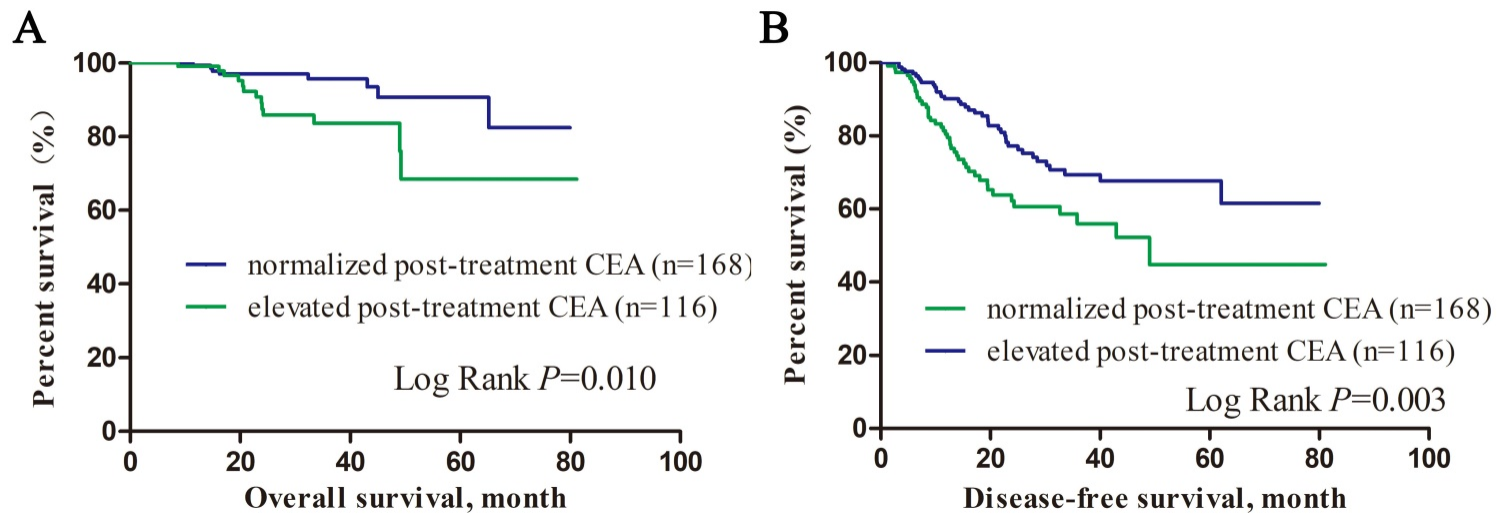
Kalan-Meier survival curves of overall survival and disease-free survival according to post-treatment CEA level.

We divided patients into subgroups according to their post-treatment CEA level. As showed in Figure 1, patients with normalized post-treatment CEA level had better overall survival (*P=*0.010) and disease-free survival (*P=*0.003) than those still with elevated CEA level after neoadjuvant therapy. Cox proportional hazard regression was used to estimate the survival predictors in LARC patients with elevated pre-treatment CEA level. Multivariate analysis showed that low albumin level (HR=0.937, 95% CI: 0.893-0.983, *P=*0.007), nerve infiltration (HR=4.160, 95% CI: 1.176-14.718, *P=* 0.027), tumor deposit (HR=3.851, 95% CI: 1.094-13.552, *P=*0.036), higher post-treatment CEA level (HR=1.042, 95% CI: 1.017-1.067, *P=*0.001) were independent unfavored predictors for overall survival. Low albumin level (HR=0.952, 95% CI: 0.914-0.992, *P=*0.019), nerve infiltration (HR=2.740, 95% CI: 1.396-5.376, *P=* 0.003), tumor deposit (HR=3.236, 95% CI: 1.738-6.026, *P<*0.001), higher pre-treatment CEA level (HR=1.004, 95% CI: 1.000-1.007, *P=*0.039) were independent unfavored predictors for disease free survival (Table 4).

## Discussion

In this study, it was revealed that post/pre-treatment CEA ratio predicted TRG and tumor downstage in LARC patients received neoadjuvant therapy and higher post-treatment CEA predicted unfavored prognosis in LARC with elevated pre-treatment CEA. Our results provided with evidence for the predictive value of CEA decline on tumor regression of LARC patients.

CEA is a highly cost-effective tumor marker for rectal cancer and its value in predicting prognosis of colorectal cancer patients had been proved 16. However, its association with tumor regression to neoadjuvant chemotherapy was still not clear. Hu H 10 and Kim J.Y 11 recommended an exponential CEA decrease model found that exponential CEA decrease was associated with pathological complete response after neoadjuvant therapy. Another study which recruited 159 patients with elevated pre-treatment CEA suggested that CEA normalization during pre-operative chemotherapy was a strong predictor for pCR 17, which was consistent with the conclusion of Yang, KL 18. In our study, we used post/pre-treatment CEA ratio as a new index to estimate the change of CEA during neoadjuvant treatment and it was suggested that lower CEA ratio was an independent predictor for favor tumor regression in terms of TRG 0/1 and tumor downstage. All these above results support that CEA level was an effective index of tumor burden for colorectal patients with elevated serum CEA. The abnormal CEA level often indicated residual tumor after neoadjuvant surgery, which would provide additional information when we dealt with radiographic remission lesions.

Higher post-treatment CEA predicted poor overall survival for LARC patients in our study. Huh, J.W et al. also demonstrated that post-treatment CEA was an independent risk factor for overall survival of patients who underwent chemoradiotherapy and total mesorectal excision 19. Patients with CEA normalization may have a better response of neoadjuvant therapy and then longer survival. Chung, M.J. et al analyzed a cohort with 104 LARC patients and conclude that CEA normalization during chemoradiotherapy was associated favor prognosis^20^, although patients with elevated pre-treatment CEA were not analyzed in subgroups. Sung, S. et al divided 110 patients with clinical T3/T4 or node positive disease underwent neoadjuvant chemoradiotherapy and total mesorectal resection into 3 groups (group A: pre-treatment CEA≤3.2ng/ml, group B: pre-treatment CEA>3.2ng/ml and post-treatment CEA≤2.8ng/ml, group C: pre-treatment CEA>3.2ng/ml and post-treatment CEA>2.8ng/ml). The 3-year disease-free survival of group A (82.5%) was comparable to that of group B (89.5%), and both were better than group C (55.1%, *P=*0.001)^21^. Above all, we could conclude that the decline of CEA during neoadjuvant therapy predicted patients' favor prognosis, which proved the survival benefit of pre-operative treatment and provides information for precise surveillance strategy.

There were some limitations in this study. Rectal cancer patients with metastatic disease were excluded, the predictive value of CEA change for tumor regression in palliative chemotherapy was not defined. Furthermore, this was a single-center retrospective study, selective bias was inevitable. Further perspective research was still needed. In conclusion, our study suggested that post/pre-treatment CEA ratio help predicting tumor regression in term of TRG and tumor downstage for LARC patients with elevated pre-treatment CEA and higher post-treatment CEA predicted poor overall survival.

## Figures and Tables

**Table 1:** Characteristics of LARC patients with elevated pre-treatment CEA according to TRG and tumor downstage

Characteristic	All patients	TRG 0/1	TRG 2/3	*P* value	Downstage	No Downstage	*P* value
Gender				0.901			0.31
Male	200(70.4)	77(70.0)	123(70.7)		134(72.4)	66(66.7)	
Female	84(29.6)	33(30.0)	51(29.3)		51(27.6)	33(33.3)	
Age	59(20-81)	58(27-78)	59.5(20-81)	0.478	59(20-81)	54(25-74)	
BMI (kg/m^2^)	22.55±3.12	22.69±3.07	22.46±3.17	0.538	22.78±3.03	22.11±3.26	0.088
Diabetes				0.35			0.357
Yes	23(8.1)	11(10.0)	12(6.9)		17(9.2)	6(6.1)	
No	261(91.9)	99(90.0)	162(93.1)		168(90.8)	93(93.9)	
Albumin (G/L)	40.56±4.76	40.63±4.41	40.52±4.99	0.85	40.25±4.99	41.16±4.27	0.125
Ca199				0.315			0.87
>37ng/ml	24(8.5)	7(6.4)	17(9.8)		16(8.6)	8(8.1)	
≤37ng/ml	260(91.5)	103(93.6)	157(90.2)		169(91.4)	91(91.9)	
Tumor Location				0.502			0.661
Upper Rectum	31(10.9)	9(8.2)	22(12.6)		19(10.3)	12(12.1)	
Middle Rectum	105(37.0)	42(38.2)	63(36.2)		66(35.7)	39(39.4)	
Lower Rectum	148(52.1)	59(53.6)	89(51.1)		100(54.0)	48(48.5)	
cTNM				0.664			<0.001
II	42(14.8)	15(13.6)	27(15.5)		15(8.1)	27(27.3)	
III	242(85.2)	95(86.4)	147(84.5)		170(91.9)	72(72.7)	
Histopathology				0.986			0.057
Classic Adenocarcinoma	266(93.7)	103(93.6)	163(93.7)		177(95.7)	89(89.9)	
Mucinous Adenocarcinoma	18(6.3)	7(6.4)	11(6.3)		8(4.3)	10(10.1)	
Tumor Differentiation				0.498			0.001
Grade 1/2	251(88.4)	99(90.0)	152(87.4)		172(93.0)	79(79.8)	
Grade 3/4	33(11.6)	11(10.0)	11(12.6)		13(7.0)	20(20.2)	
Preoperative Radiology				0.025			0.088
Yes	111(39.1)	52(47.3)	59(33.9)		79(42.7)	32(32.3)	
No	173(60.9)	58(52.7)	115(66.1)		106(57.3)	67(67.7)	
Post-treatment CEA (ng/mL)	4.23 (0.50-173.80)	2.77 (0.50-49.77)	4.91 (0.51-173.80)	<0.001	3.75 (0.50-173.80)	4.51 (0.50-72.88)	0.043
Pre-treatment CEA (ng/L)	11.87 (5.02-731.31)	11.12 (5.05-203.38)	12.58 (5.02-731.31)	0.154	11.26 (5.02-731.31)	12.96 (5.04-222.41)	0.711
Post/pre-treatment CEA Ratio	0.31 (0.01-2.55)	0.23 (0.02-1.48)	0.37 (0.01-2.55)	<0.001	0.28 (0.01-1.71)	0.38 (0.03-2.55)	0.025

Abbreviations: LARC: Local Advanced Rectal Cancer, CEA: Carcinoembryonic Antigen, TRG: Tumor Regression Grade, BMI: Body Mass Index

**Table 2:** Logistic Regression Model on TRG 0/1 for LARC patients

Risk Factors	Univariate Logistic Regression			Multivariate Logistic Regression		
	OR	95% CI	P Value	OR	95% CI	P Value
Preoperative radiology (No vs Yes)	1.748	1.072-2.849	0.025	1.372	0.643-2.924	0.413
Post-treatment CEA (ng/ml)	0.896	0.84-0.956	0.001	0.936	0.883-0.993	0.027
Post/pre-treatment CEA Ratio	0.21	0.089-0.495	<0.001	0.289	0.106-0.788	0.015

Abbreviations: TRG: Tumor Regression Grade, LARC: Local Advanced Rectal Cancer, CEA: Carcinoembryonic Antigen

**Table 3:** Logistic Regression Model on tumor downstage for LARC patients

Risk Factors	Univariate Logistic Regression			Multivariate Logistic Regression		
	OR	95% CI	*P* Value	OR	95% CI	*P* Value
Age	1.029	1.008-1.050	0.005	1.031	1.008-1.054	0.007
Preoperative radiology (No vs Yes)	1.560	0.935-2.604	0.089	N/A	N/A	N/A
cTNM (III vs II)	4.250	2.134-8.462	<0.001	4.405	2.150-9.025	<0.001
Tumor Differentiation (G1/2 vs. G3/4)	3.350	1.586-7.072	0.002	2.757	1.238-6.137	0.013
Post-treatment CEA (ng/ml)	0.988	0.969-1.008	0.229	N/A	N/A	N/A
Post/pre-treatment CEA Ratio	0.445	0.223-0.887	0.021	0.393	0.187-0.829	0.014

Abbreviations: LARC: Local Advanced Rectal Cancer, CEA: Carcinoembryonic Antigen, N/A: Not Applicable

**Table 4:** Cox proportional hazard regression model for overall survival and disease free survival in LARC patients

Characteristic	Overall Survival			Disease Free Survival		
	Hazard Ratio	95% CI	*P* value	Hazard Ratio	95% CI	*P* value
Albumin (g/L)	0.937	0.893-0.983	0.007	0.952	0.914-0.992	0.019
CA19-9 (≤37 U/mL vs >37 U/mL)	0.771	0.178-3.339	0.728	1.392	0.652-2.973	0.393
Vascular infiltration (No vs Yes)	3.720	0.859-16.106	0.079	0.957	0.398-2.300	0.922
Nerve infiltration (No vs Yes)	4.160	1.176-14.718	0.027	2.740	1.396-5.376	0.003
Tumor deposit (No vs Yes)	3.851	1.094-13.552	0.036	3.236	1.738-6.026	<0.001
ypTNM	0.681	0.388-1.194	0.180	1.317	0.974-1.782	0.074
Pre-treatment CEA (ng/ml)	1.003	0.996-1.011	0.405	1.004	1.000-1.007	0.039
Post-treatment CEA (ng/ml)	1.042	1.017-1.067	0.001	1.009	0.996-1.023	0.186

Abbreviations: LARC: Local Advanced Rectal Cancer, CEA: Carcinoembryonic Antigen
